# Three novel and the common Arg677Ter RP1 protein truncating mutations causing autosomal dominant retinitis pigmentosa in a Spanish population

**DOI:** 10.1186/1471-2350-7-35

**Published:** 2006-04-05

**Authors:** María José Gamundi, Imma Hernan, María Martínez-Gimeno, Miquel Maseras, Blanca García-Sandoval, Carmen Ayuso, Guillermo Antiñolo, Montserrat Baiget, Miguel Carballo

**Affiliations:** 1 Servicio de Laboratorio, Hospital de Terrassa. Ctra. Torrebonica s/n 08227 Terrassa, Barcelona, España; 2 Servicio de Oftalmología, Hospital de Terrassa. Ctra. Torrebonica s/n 08227 Terrassa, Barcelona, España; 3 Servicio de Oftalmología, Fundación Jiménez Díaz, Madrid; 4 Servicio de Genética, Fundación Jiménez Díaz, Madrid; 5 Servicio de Genética, Hospital Virgen del Rocío, Sevilla, España; 6 Servicio de Genética, Hospital de la Santa Creu i Sant Pau, Barcelona, España

## Abstract

**Background:**

Retinitis pigmentosa (RP), a clinically and genetically heterogeneous group of retinal degeneration disorders affecting the photoreceptor cells, is one of the leading causes of genetic blindness. Mutations in the photoreceptor-specific gene RP1 account for 3–10% of cases of autosomal dominant RP (adRP). Most of these mutations are clustered in a 500 bp region of exon 4 of RP1.

**Methods:**

Denaturing gradient gel electrophoresis (DGGE) analysis and direct genomic sequencing were used to evaluate the 5' coding region of exon 4 of the RP1 gene for mutations in 150 unrelated index adRP patients. Ophthalmic and electrophysiological examination of RP patients and relatives according to pre-existing protocols were carried out.

**Results:**

Three novel disease-causing mutations in RP1 were detected: Q686X, K705fsX712 and K722fsX737, predicting truncated proteins. One novel missense mutation, Thr752Met, was detected in one family but the mutation does not co-segregate in the family, thereby excluding this amino acid variation in the protein as a cause of the disease. We found the Arg677Ter mutation, previously reported in other populations, in two independent families, confirming that this mutation is also present in a Spanish population.

**Conclusion:**

Most of the mutations reported in the RP1 gene associated with adRP are expected to encode mutant truncated proteins that are approximately one third or half of the size of wild type protein. Patients with mutations in RP1 showed mild RP with variability in phenotype severity. We also observed several cases of non-penetrant mutations.

## Background

Retinitis pigmentosa (RP) is a common inherited retinopathy that affects more than one million people worldwide. Clinical findings in RP include night blindness, loss of peripheral vision and progressive degeneration of the retina that usually culminates in severe visual impairment or complete blindness [1]. The disease is characterized by abnormal or absent responses on electroretinography (ERG) and is associated with retinal atrophy, bone spicule-like pigmentary deposits and attenuation of retinal vessels. RP can be inherited in autosomal dominant, autosomal recessive, X linked [2-5] or digenic forms [6]. Several genes have been identified by linkage studies or candidate gene screening for each of these RP types [7] , although the majority remain unknown [8]. Autosomal dominant retinitis pigmentosa (adRP) accounts for 25–30% of all Spanish RP patients (unpublished data). Mutations in genes with specific expression in the retina causing adRP have been reported [2-8]: rhodopsin (RHO), peripherin/RDS, NRL, CRX, RP1 and FSCN2 [9]. The major contribution in a Spanish population is due to rhodopsin mutations (19.5 %), with the rest of the specific genes expressed in the retina less than 4% [unpublished results] More recently, mutations in the ubiquitously expressed pre-mRNA splicing factor genes PRPF3, PRPF8, PRPF31 or in the IMPDH1 gene have also been associated with adRP [10-13]. The contribution of mutations in these genes is above 8% in a Spanish population [unpublished results].

The RP1 gene has been localized to the pericentric region of chromosome 8 [14]. The RP1 gene has four exons giving rise to a mRNA of about 7 kb, which is predicted to encode a protein of 2156 amino acids in length [15-17]. Immunofluorescence analysis carried out in human and mouse retinas revealed that RP1 proteins are specifically localized to the connecting cilia of rod and cone photoreceptors [18]. Mouse models of the RP1 form of RP have been generated by targeted disruption of the mouse ortholog (Rp1) of human RP1. Studies carried out in these mouse models demonstrate that the Rp1 protein is required for normal morphogenesis of the photoreceptor outer segment [19]. Recent studies indicate that RP1 is required for the correct orientation and higher order stacking of outer segment discs [20]. Furthermore, the RP1 protein has been reported as a microtubule-associated protein (MAP) forming part of the photoreceptor axoneme [21]

Screening for mutations in RP1 has been carried out in different adRP populations [22-27]. Most of the mutations in RP1 causing adRP are single nucleotide substitutions that produce a premature stop codon or insertion/deletion changes predicting a truncated protein [23]. These mutations are mostly located in the 5' coding region of the 4 kb long exon 4 of the RP1 gene.

## Methods

Informed consent was obtained from all subjects who participated in the study and the research adhered to the tenets of the Declaration of Helsinki.

### Ophthalmological and electrophysiological studies

A complete ophthalmic examination was performed in all patients. The examination consisted of best corrected visual acuity with Snellen optotypes, computerised perimetry (recorded with the OCTOPUS 500) and biomicroscopy and fundus examination after pupillary dilatation. Electroretinograms (ERG) were performed according to the standard testing protocols proposed by ISCEV [28].

### Polymerase Chain Reaction (PCR)

Genomic DNA was prepared from peripheral blood lymphocytes using QIAmp DNA Blood Mini Kit (Qiagen, Valencia, CA). The coding regions of exon 4 of the RP1 gene were amplified using primers (Table 1). One PCR primer in each pair included a 40-base GC-rich segment ("GC-clamp") attached to its 5' end to facilitate the detection of mutations by denaturing gradient gel electrophoresis (DGGE). PCR reactions were performed in a 50 μl volume of buffer (20 mM Tris-HCl pH 8.55, 16 mM (NH)_2_SO_4_, 1.5 mM MgCl_2 _150 μg/ml BSA, 10% DMSO) containing 50–200 ng of human genomic DNA, 25 picomols of each primer, 10 nanomols of each deoxyribonucleoside triphosphate, and 1.5 units of Taq polymerase. Incubation was performed for 40 cycles consisting of 30 seconds at 94°C, 30 seconds at 58°C and 30 seconds at 72°C; followed by 5 minutes at 94°C and 5 minutes at 72°C. Electrophoresis of 8 μl of final PCR reaction volume was performed on 1.5% agarose gel to test the amplification reaction.

**Table 1:** Primers and DGGE screening conditions

Primers*	**cDNA Sequence**	**Fragment size (bp)**	Gradient %**	**Running T (°C)**
Forward 5'-GTAATAACTCTGGAACTGACAA-3'	1998–2308	350	40–70	50
Reverse 5'-**GC**-GGACTATCTGAACTTTGCACTA-3'				
Forward 5'-CAGGTATCAAGATGGACAGC-3'	2197–2461	304	40–70	50
Reverse 5'-**GC**-TGAACCTTGGAATTTTGAGTAG-3'				

* **(GC) **= 5'-CGCCCGCCGCGCCCCGCGCCCGGCCCGCCGCCCCCGCCCG-3' ** 100% denaturant = 7M urea and 40% (v/v) formamide in TAE buffer

### Mutation detection

Mutation analysis of the coding region of exon 4 of the RP1 gene was carried out by DGGE [29,30]. Table 1 shows the electrophoretic conditions, including the running temperature and the denaturing gradient of the formamide/urea concentration range for each different PCR product. The DNA from the PCR fragment containing the R677X mutation was digested with the endonuclease TaqI (Roche, Barcelona, Spain), according to the manufacturer's specifications. For DNA sequencing, PCR products were purified using the QIAquick Gel Extraction Purification Kit (Qiagen). DNA sequencing was carried out using the OpenGene automated DNA sequencing system from Visible Genetics and Thermo Sequenase Cy5.5 Dye Terminator Cycle Sequencing Kit (Amersham Pharmacia Biotech, Barcelona, Spain).

## Results

**Figure 1:**
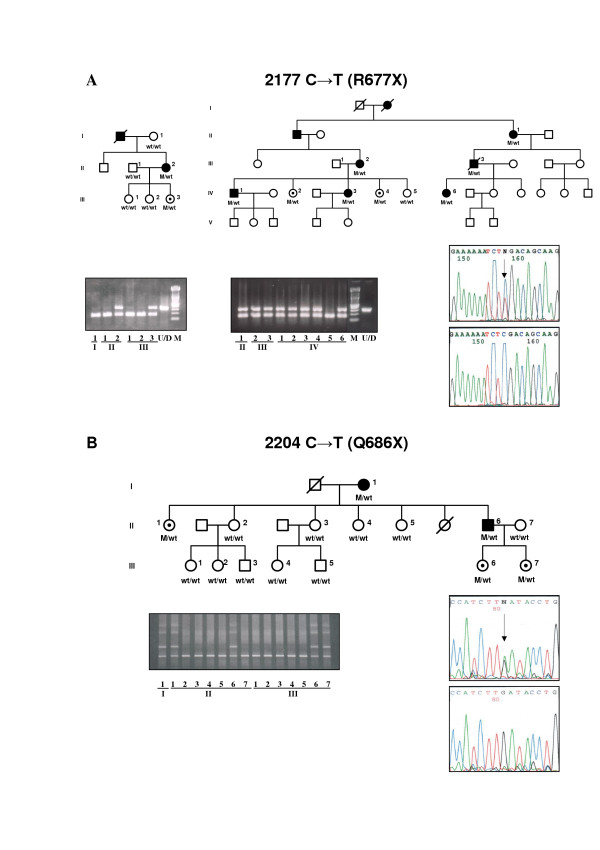
**Mutation in the RP1 gene due to nucleotide substitution**. **A. **Pedigree, restriction analysis, and direct sequencing of Spanish families showing the R677X mutation. The 2177 C→T substitution abolishes the TaqI restriction site. M is the DNA marker consisting of a 100 bp ladder and U/D is the undigested DNA PCR fragment. **B**. Pedigree, DGGE and direct sequencing of the family carrying the Q686X mutation in the RP1 gene. Affected individuals, asymptomatic carriers and non-carriers of the mutation are represented by solid symbols, symbols with an internal dot and open symbols, respectively.

**Figure 2:**
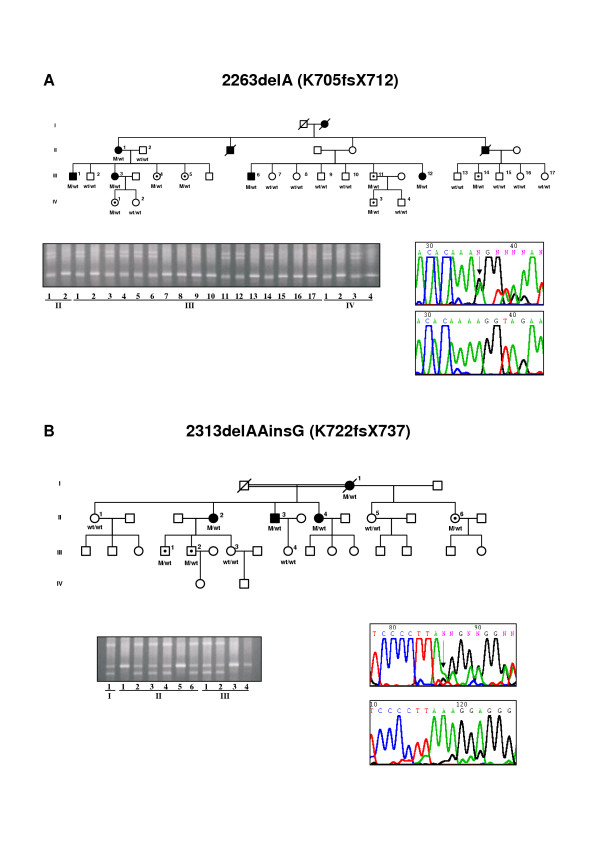
**Mutation in the RP1 gene by A deletions**. Pedigree, DGGE and direct sequencing of Spanish families showing K705fsX712 **(A) **and K722fsX737 **(B) **in the RP1 gene. Affected individuals, asymptomatic carriers and non-carriers of the mutation are represented by solid symbols, symbols with an internal dot and open symbols, respectively.

**Figure 3:**
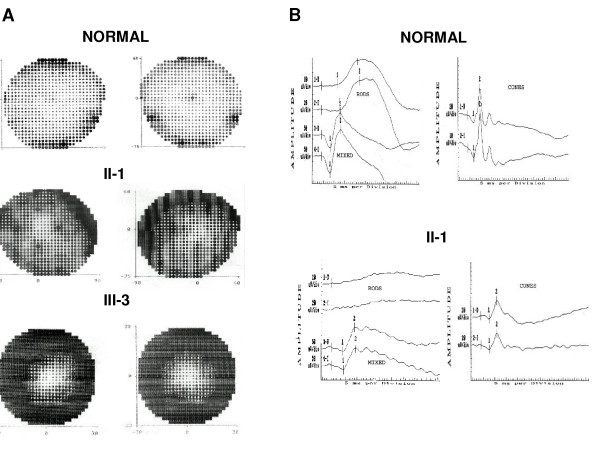
**Intrafamilial variability of disease expression in the K705fsX712 RP1 mutation**. **A**. Visual field tests recorded in a normal individual, patient II-1 at the age of 72 years and III-3 at the age of 40, carrying the mutation. **B**. Electroretinographic recording in a normal individual and in patient II-1 at the age of 72 years still showing response of the rods and cones while in patient III-3 the ERG was abolished (not shown).

We screened Spanish families with adRP for mutations between c-DNA positions 1998 and 2639 (GenBank: AF141021) in exon 4 of the RP1 gene. Three PCR-amplified fragments from DNA of the 150 index patients of adRP Spanish families and 100 controls were analyzed by DGGE. Five different electrophoretic patterns were identified. Direct DNA sequencing revealed four mutations that predict truncated proteins and one missense mutation, Thr752Met (Table 2). These sequence changes were not observed in the controls. The common mutation Arg677Ter was detected in two index cases. Because this mutation abolishes the TaqI endonuclease restriction site, restriction analysis was performed in the members of these two families (Figure 1A). Co-segregation analysis carried out in the adRP families demonstrated that the four mutations generating truncated proteins were carried by all the affected members of the family (Figures 1 and 2). However, the missense mutation Thr752Met was not carried by several RP patients, though it was present in some unaffected individuals of the family (data not shown). The two frameshift mutations, 2263delA and 2313delAAinsG produce 7 and 15 altered codons respectively, before a premature stop. The Spanish families with adRP caused by mutations in the RP1 gene showed a mild form of RP. Affected members of these families generally had a late onset of night blindness and slow loss of visual acuity and visual fields (Table 3). ERGs of the patients were usually abolished (Tables 4 and 5) but in some individuals, even at an advanced age, a diminished signal was recordable (Figure 3). This mild clinical expression may explain the familial heterogeneity and the fact that nearly half of the individuals with a mutation in RP1 were asymptomatic (Table 2). Furthermore, incomplete penetrance previously reported in RP1 forms could also be present in these families (Figures 1 and 2).

**Table 2:** Mutations detected in the Spanish adRP families

**Exon**	**DNA change**	**Mutation**	**Symptomatic carriers**	**Asymptomatic carriers**	**Total carriers**
4	2177 C > T (CGA→TGA)	Arg677Ter	5	3	8
4	2204C > T (CAA→TAA)	Gln686Ter	2	2	4
4	2263delA	Lys705fsX712	6	5	11
4	2313delAAinsG	Lys722fsX737	3	3	6
4	2403C > T (ACG→ATG)	Thr752Met*	1	-	1

*This mutation does not appear to be pathogenic.

**Table 3:** Clinical features of Spanish families with RP1 mutations

**Mutation**	**Family members**	**Age (years)**	**Onset of NB**	**Onset ↓VFC**	**Onset ↓VA**	**Current VF**	**Current VA**	**Current ERG**	**Funduscopy**
K722fsX737	I-1	85	60	65	None	Not done	Not done	Not done	Typical RP
	II-2	59	50	49	None	10°central	0.6 BE	Not detectable	Typical RP
	II-3	58	43	46	50	10°central	< < 0.1 BE	Not detectable	Typical RP + CME
	II-4	56	37	54	None	10°central	0.5 BE	Not detectable	Typical RP

Q686X	I-1	83	69	74	69	10°central	0.2 BE	R/M nd. & ↓↓	Typical RP
	II-1	60	None	None	None	Normal	1.0 BE	Normal	Normal
	II-6	54	30	45	25	10°central	0.3 BE	R/M & ↓↓ C N	Typical RP
	III-6	29	None	None	None	Normal	1.0 BE	Normal	Normal
	III-7	26	None	None	None	Normal	1.0 BE	Normal	Normal

K705fsX712	II-1	73	None	None	None	PC up VF	0.7/0.6	R nd & M/C↓	Normal
	III-1	46	Unknown	Unknown	Unknown	PC	Not done	Not done	Typical RP
	III-3	42	30	35	None	20°R/10°L	0.8/1	Not detectable	Typical RP
	III-4	40	None	None	None	Normal	1.0 BE	Normal	Normal
	III-5	39	None	None	None	Normal	1.0 BE	Normal	Normal
	III-6	49	Unknown	Unknown	Unknown	PC	Not done	Not done	Typical RP
	III-11	41	None	None	None	Normal	1.0 BE	Normal	Normal
	III-12	38	Unknown	Unknown	Unknown	PC	Not done	Not done	Typical RP
	III-14	39	None	None	None	Normal	1.0 BE	Normal	Normal
	IV-1	11	None	None	None	Normal	1.0 BE	Normal	Normal

***BE**: Both Eyes; **ERG**: Electroretinogram; **CME**: Cystoid Macular edema; **NB**: Night blindness; **PC upVF**: Peripheral constriction of upper visual field; **R nd & M/C↓**: Not detectable rod responses, impaired amplitude and delayed response of mixed and cones; **R/M ↓↓ & C N**: Impaired amplitude and delayed response of rods and mixed, normal cone response; **R/M nd. & C↓↓**: Not detectable rod and mixed responses, impaired amplitude and delayed response of cones; **Typical RP**: Pale discs, attenuated vessels, bone-spicule intraretinal pigmentation; **VA**: Visual acuity; **VF**: Visual field; **VFC**: Visual field constriction*.

**Table 4:** ERG values of Spanish families with RP1 mutations

		**RODS**		**MIXED (ROD-CONES)**				**CONOS**		**30 Hz Flicker**	
**INDIVIDUAL**	**EYE**	**b-wave AMPL.**	**b-wave IMP.T**	**a-wave AMPL**	**a-wave IMP.T.**	**b-wave AMPL.**	**b-wave IMP.T**	**b-wave AMP.**	**b-wave IMP.T**	**b-wave AMPL**	**b-wave IMP.T**

**Q686X**											
I-1	R	ND	ND	ND	ND	ND	ND	50	36	NV	NV
	L	ND	ND	ND	ND	ND	ND	30	37	NV	NV
II-1	R	260	109	378	23	874	49	218	30	131	29
	L	230	105	363	23	833	48	238	31	144	29
II-6	R	ND	ND	ND	ND	ND	ND	ND	ND	ND	ND
	L	ND	ND	ND	ND	ND	ND	ND	ND	ND	ND
III-6	R	110	93	158	22	389	44	142	31	97	27
	L	157	108	213	22	441	44	140	31	65	25
III-7	R	116	99	197	22	491	45	154	30	68	27
	L	137	94	170	22	412	45	NV	NV	NV	NV

**K705fsX712**											
II-1	R	ND	ND	39	24	267	49	85	34	38	32
	L	ND	ND	51	26	246	52	76	35	63	33
III-1	R	-	-	-	-	-	-	-	-	-	-
	L	-	-	-	-	-	-	-	-	-	-
III-3	R	ND	ND	ND	ND	ND	ND	ND	ND	ND	ND
	L	ND	ND	ND	ND	ND	ND	ND	ND	ND	ND
III-4	R	177	93	198	21	492	51	198	29	148	27
	L	187	93	199	21	527	49	230	30	122	27
III-5	R	186	104	221	22	556	47	166	30	58	28
	L	209	109	219	21	500	47	168	30	75	28
III-6	R	-	-	-	-	-	-	-	-	-	-
	L	-	-	-	-	-	-	-	-	-	-
III-11	R	165	103	274	22	500	46	101	32	77	30
	L	169	106	299	23	563	46	104	31	83	29
III-12	R	-	-	-	-	-	-	-	-	-	-
	L	-	-	-	-	-	-	-	-	-	-
III-14	R	170	100	266	21	705	49	207	29	127	29
	L	166	103	195	21	570	48	204	29	100	29
IV-1	R	106	86	150	21	393	45	152	29	64	26
	L	138	91	191	21	491	46	193	29	80	27

**K722fsX737**											
I-1	R	-	-	-	-	-	-	-	-	-	-
	L	-	-	-	-	-	-	-	-	-	-
II-2, II-3, II-4	R	ND	ND	ND	ND	ND	ND	ND	ND	ND	ND
	L	ND	ND	ND	ND	ND	ND	ND	ND	ND	ND

**ND**: Not detectable; **NV**: Not valid; -:Not done or not available

**Table 5:** Normal values of ERG

**RODS**		**MIXED (ROD-CONES)**				**CONOS**		**30 Hz Flicker**	
**b-wave AMPL.**	**b-wave IMP.T.**	**a-wave AMPL.**	**a-wave IMP.T.**	**b-wave AMPL.**	**b-wave IMP.T.**	**b-wave AMP.**	**b-wave IMP.T.**	**b-wave AMPL.**	**b-wave IMP.T.**

100 – 350	70 – 110	150 – 360	25 – 30	380 – 750	35 – 55	85 – 225	26 – 31	20 – 140	24 – 35

**AMPL. **(Amplitude) = μV **IMP.T. **(Implicit Time) = ms

## Discussion

Mutations in the RP1 gene account for 5–10% of adRP in American and British populations [24]. RP1 is the second most frequently mutated gene causing adRP, the first being rhodopsin (20–25% of cases). Most disease-causing mutations in the RP1 gene are predicted to give rise to a truncated protein. These mutations tend to be clustered in a region within the first third of exon 4 of the RP1 gene, suggesting that truncation of the extreme C-terminal end of the RP1 protein does not cause adRP.

Except for a few missense mutations of uncertain pathogenicity, the dominant mutations in the RP1 gene have been found in a region extending from codons 658 to 1053 in exon 4, although most are clustered between codons 658–872 [24]. These mutations are mainly single nucleotide changes, insertion or deletion sequences leading to premature stop codons that presumably encode truncated proteins of approximately one third the length of the wild type protein [22-27]. We screened for mutations within the region extending from codons 616–879 in exon 4 of the RP1 gene in Spanish families with adRP. By only screening part of the RP1 gene, we are probably missing novel mutations in other parts of the gene, which may underestimate the total contribution of RP1 to adRP in the Spanish population analyzed.

Four disease-causing mutations in the RP1 gene were found in five of 150 (3.5%) adRP families. The Arg677Ter mutation was detected in two unrelated families. This mutation has been reported in apparently independent families from British, Chinese and North American populations [17,18,26] suggesting a hotspot mutation. Genotype analysis showed no linkage between the two Spanish families carrying the Arg677Ter mutation, supporting the suggestion that this mutation is a hotspot rather than an ancestral mutation in the RP1 gene [23]. Further support for this hotspot hypothesis was recently provided in a report of a *de novo *Arg677Ter RP1 mutation [31]. The mutations in the RP1 gene associated with adRP which we found in the Spanish population are expected to result in premature termination of the protein. Two types of mutations in the RP1 sequence were found. One mutation was produced by a single nucleotide change C to T leading to a premature stop codon (Figure 1). The second type was due to insertion or deletion of nucleotides in regions with multiple series of A residues in the DNA sequence of the RP1 gene (Figure 2). These sequences could cause DNA slippage due to mismatch of the two DNA strands during replication, thereby generating mutations [32]. This mechanism could explain the clustered mutations in a region rich in series of A sequences within exon 4 of RP1 which result in truncated mutant proteins.

Nonsense mutations in mammalian genes generally lead to unstable mRNA molecules with very little or no translated protein [33]. However, this mechanism seems to be less probable for the nonsense mutations in the last exon of the gene [33]. This is the case in the RP1 mutations in exon 4, which are probably translated as truncated proteins. Recent experiments using lymphoblastoid cell lines from patients with the Arg677Ter mutation in illegitimate transcription assays detected mRNA carrying the Arg677Ter mutation [20] , suggesting that truncated RP1 proteins may be produced in the retina. Thus, the truncated proteins generated in the RP1 mutants may exert a deleterious effect on the photoreceptor cell. However, studies carried out in murine models with Rp1 truncated proteins showed that these proteins appear to be non-functional and do not seem to exert a dominant negative effect in heterozygous mice [20].

Sequence variations have also been detected in the screening of the RP1 gene. Most of the missense mutations reported in the RP1 gene do not cause adRP [24]. We detected the Thr752Met mutation in one adRP family. This mutation was not detected in two RP patients but it was present in unaffected members of the family. Furthermore, the clinical RP phenotype of the affected members of this family resulted in a more severe disease expression than in the patients with RP caused by a mutation in the RP1 gene. The Thr752Met mutation is unlikely, therefore, to be associated with adRP.

Recent studies using murine models of RP have contributed to the understanding of the function of the Rp1 protein [19-21]. The Rp1 protein is required in normal morphogenesis and in the correct stacking of the outer segment discs of the photoreceptors. The Rp1 protein may also play a role in the transport of rhodopsin to the outer segments [18,19]. The N-terminus of the human RP1 protein shares significant homology with doublecortin (DCX), a mutant of which is involved in a cerebral cortex abnormality [34]. The DCX region homologous to RP1 is known to interact with microtubules [35]. Recently, RP1 has also been shown to be a microtubule-associated protein forming part of the larger class of MAP proteins, whose dysfunction is associated with degenerative diseases [21].

**Figure 4:**
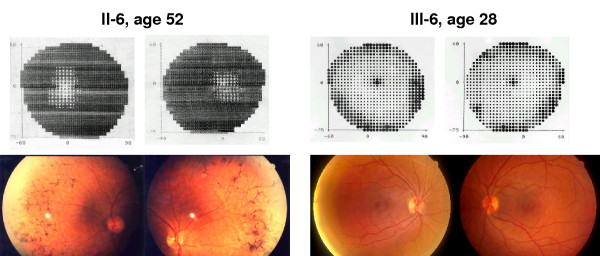
**Intrafamilial variability of disease expression in the Q686X RP1 mutation**. Comparison of visual field test and ocular fundus of patient II-6 with the asymptomatic III-6 member of the family, both carrying the mutation in the RP1 gene.

The mutations detected in RP1 patients probably generate a deficiency in the function of RP1 in the retina. The majority of the patients who are heterozygous for the RP1 mutation show a type 2 autosomal dominant RP phenotype, with a relatively late onset of night blindness by the third or fourth decade of life [24,25]. Patients who are homozygous for a mutation in RP1, however, show a more severe RP phenotype [16]. Nevertheless, considerable variation may exist within the same family in the age at which the clinical disease is manifested. In some of the Spanish families we detected asymptomatic carriers of mutations in RP1 (Figure 4) whereas younger siblings were affected. Some of these asymptomatic individuals carrying the mutation even showed a normal or near normal ERG in the third or fourth decade of life. In such adRP families with late onset or slow degeneration, screening for mutations in all at-risk members would seem essential.

## Conclusion

In conclusion, the contribution to adRP of RP1 mutations detected in the clustered mutation region of the RP1 gene associated with adRP was 3.3% in a Spanish population. We also detected the common mutation Arg677Ter, which has been reported in other ethnic groups. The individuals carrying mutations in the RP1 gene showed a mild RP phenotype with late onset of the disease or even remaining asymptomatic, suggesting incomplete penetrance in some families. All the mutations detected in the RP gene associated with ADRP are expected to encode truncated proteins.

## Competing interests

The author(s) declare that they have no competing interests.

## Authors' contributions

MJG, MMG and IH carried out the molecular genetic analysis, were involved in drafting the manuscript and revised the paper. MM and BGS were involved in ophthalmic examination of the patients. CA, GA anb MB contributed to the clinical genetic classification of the patients and families, and provided the DNA samples. MC contributed to conception and design of the study, analysed and interpreted data and wrote the paper.

## Pre-publication history

The pre-publication history for this paper can be accessed here:


